# High Body Roundness Index Is Associated With Unhealthy Sleep Patterns: Insights From NHANES (2007–2014)

**DOI:** 10.1002/brb3.70224

**Published:** 2024-12-31

**Authors:** Pingchuan Liu, Yuding Luo, Xing He, Jiali Zhang, Fanzhou Ren, Bingyang Zhang, Bo Zheng, Jian Wang

**Affiliations:** ^1^ Department of Neurology The Affiliated Hospital Southwest Medical University Luzhou China; ^2^ Department of Neurology Ya'an People's Hospital Ya'an China; ^3^ Department of Neurosurgery The Third Hospital of Mianyang Sichuan Mental Health Center Sichuan China; ^4^ North Sichuan Medical College Nanchong China

**Keywords:** anthropometric indices, body roundness index (BRI), fat distribution, obesity, sleep patterns

## Abstract

**Background:**

Substantial evidence suggests an association between obesity and sleep. However, research investigating sleep patterns in relation to novel anthropometric indices is limited. Therefore, we conducted a cross‐sectional analysis of data from the National Health and Nutrition Examination Survey (NHANES) from 2007 to 2014 to examine the relationship between the body roundness index (BRI) and unhealthy sleep patterns.

**Objective:**

This study aimed to investigate the association between the BRI and unhealthy sleep patterns among US adults.

**Methods:**

Data were sourced from NHANES (2007–2014), including respondents aged 20 years and older. Participants were categorized into two groups based on the healthiness of their sleep patterns. The data were weighted, and multiple potential covariates were included in the analysis to provide national estimates and account for the comprehensive sampling design. A multivariable weighted logistic regression model was used, employing restricted cubic spline (RCS) curves to examine potential associations, and subgroup analyses were conducted to determine the stability of the results. Receiver operating characteristic (ROC) analysis was used to compare the diagnostic performance of BRI and body mass index (BMI) in identifying unhealthy sleep patterns.

**Results:**

In the fully adjusted multivariable logistic regression model, the prevalence odds ratio (POR) for the association between BRI and unhealthy sleep patterns was 1.09, with a 95% confidence interval (CI) of 1.07–1.10. The RCS analysis found that the nonlinear association between BRI and unhealthy sleep patterns was not significant. Subgroup and sensitivity analyses indicated a consistently positive association between high BRI and unhealthy sleep patterns across most subgroups. ROC diagnostic tests showed that BRI's effectiveness in diagnosing unhealthy sleep patterns was comparable to that of BMI, and it was not inferior to BMI in assessing certain components of sleep patterns.

**Conclusion:**

High BRI is positively associated with unhealthy sleep patterns significantly, indicating that BRI could be a promising metric for evaluating sleep health.

## Graphical abstract

This study identifies a positive association between high body roundness index (BRI) and unhealthy sleep patterns in US adults, highlighting BRI's potential as a metric for sleep health evaluation.

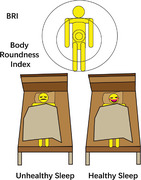

**Abbreviations**
BRI: body roundness index
CI: confidence interval
CVD: cardiovascular disease
IL‐6: interleukin‐6
MEC: mobile examination center
MET: metabolic equivalent
NHANES: National Health and Nutrition Examination Survey
OSA: obstructive sleep apnea
POR: prevalence odds ratio
RCS: restricted cubic spline
ROC: receiver operating characteristic
TNF‐α: tumor necrosis factor‐alpha
WC: waist circumference

## 1 Introduction

In Western countries, overweight and obesity have become highly prevalent issues. Obesity not only poses a serious threat to health but also significantly increases the risk of various chronic diseases, such as diabetes, cardiovascular diseases (CVDs), and obesity‐related cancers (Elmaleh‐Sachs et al. 2023). Obesity serves as a gateway to a range of other non‐communicable and communicable diseases, and without timely intervention, obese individuals may develop more complex health conditions (Frühbeck et al. 2019). Obesity is a multifactorial, chronic, and recurrent non‐communicable disease characterized by abnormal or excessive fat accumulation in the body (Busetto et al. 2024). Therefore, obesity is typically diagnosed based on body mass index (BMI) thresholds, although this approach fails to adequately account for the distribution of adipose tissue and its functional differences in obesity severity (Zapata et al. 2023). In contrast, Thomas, Bredlau, and Bosy‐Westphal (2013) proposed a novel anthropometric measure, the body roundness index (BRI), which better reflects fat distribution.

Sleep plays a vital role in maintaining and enhancing both physical and mental health. However, many people sleep less than the recommended amount and even suffer from sleep disorders (Baranwal, Yu, and Siegel 2023). Sleep disorders are closely associated with various adverse health conditions, such as diabetes, CVDs, and urological symptoms (Xin et al. 2022). Previous studies examining the relationship between sleep and obesity have often focused on limited sleep factors, such as sleep duration (Grandner et al. 2015) or sleep quality (Narcisse et al. 2018). Indicators for evaluating sleep health are multidimensional and should not be restricted to sleep duration alone; they should also include assessments of sleep quality, such as sleep trouble and difficulties in falling asleep (Knutson et al. 2017). Compared to studies using a single sleep indicator, composite sleep metrics that consider the interaction of data such as sleep duration and sleep trouble can more accurately predict disease risk (Chandola et al. 2010). For example, sleep patterns are defined by covering sleep duration, sleep difficulties, and sleep disorders (Liu and Chien 2023). However, the association between sleep patterns and BRI remains unclear. We hypothesize that a higher BRI is associated with unhealthy sleep patterns. This study aims to assess the association between BRI and sleep patterns, thereby providing new insights for identifying and preventing unhealthy sleep patterns.

## 2 Methods

### 2.1 Study Population

**FIGURE 1:**
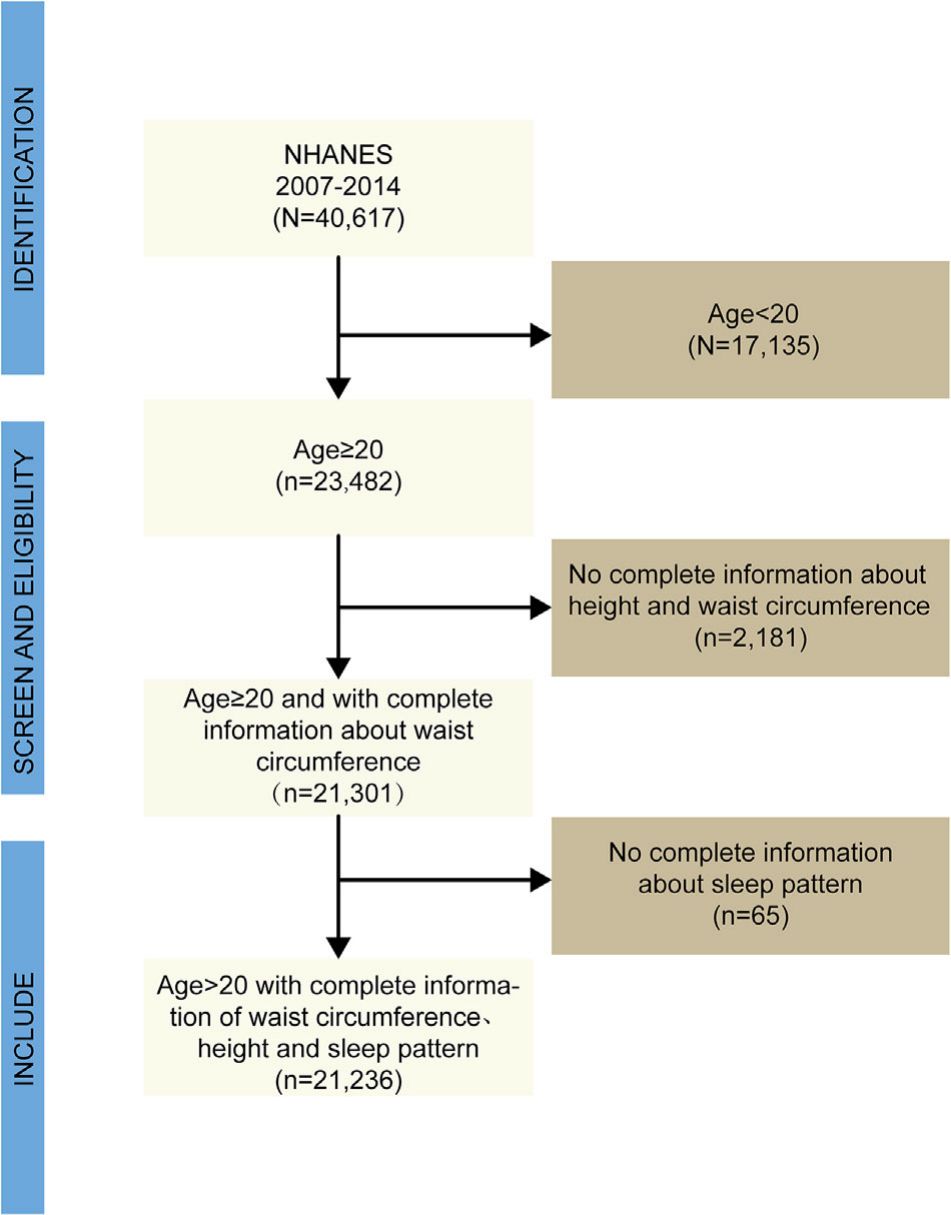
Flowchart of the population included in the final analysis. NHANES, National Health and Nutrition Examination Survey.

The National Health and Nutrition Examination Survey (NHANES) is a nationally representative, complex, stratified, multi‐stage, cross‐sectional survey in the United States, designed to investigate health and nutrition‐related risk factors within the US population (US Preventive Services Task Force et al. 2022). Since 1999, NHANES has been conducted every 2 years, with different participants in each cycle. Assessments are conducted through household interviews and mobile examination centers (MECs). The survey has been approved by the Research Ethics Review Board of the National Center for Health Statistics, and all participants provided written informed consent. This study utilized data from four NHANES cycles conducted between 2007 and 2014. Samples with incomplete questionnaire information across the four cycles were excluded from this study. The inclusion and exclusion criteria for the study are detailed in Figure 1.

### 2.2 Exposure and Outcome Definitions

The BRI was specified as the exposure factor, which primarily includes two variables used to assess obesity: height and waist circumference. Measurements of height and waist circumference were taken by medical professionals through anthropometric examinations conducted at the MEC. The outcome, sleep patterns, was composed of three factors: self‐reported sleep duration, sleep difficulties, and diagnosed sleep disorders. Sleep duration was categorized as short (<7 h per night), normal (7–9 h per night), and long (>9 h per night). Lower or higher risks were classified as 0 or 1, respectively, to calculate a cumulative score ranging from 0 to 3. A sleep score of 0 or 1–3 was classified as healthy or unhealthy sleep patterns, respectively (Lu et al. 2023):

$$\mathrm{BRI}=364.2-365.5\times \sqrt{1-\frac{{\left[\frac{WC}{2\pi }\right]}^{2}}{{\left[0.5\times h\right]}^{2}}}$$

where BRI is body roundness index; WC is waist circumference, measured in meters; and h is height, measured in meters.

### 2.3 Assessment of Covariates

Demographic data included sex, age, race, education level, marital status, and poverty income ratio (PIR). Second, smoking status was categorized as “former smoker,” “current smoker,” and “never smoker.” Alcohol use and self‐reported diseases (such as diabetes, hypertension, and CVDs) were recorded as “yes” or “no.” Metabolic equivalent (MET) was used to assess physical activity, categorized as insufficient activity (MET <600) and sufficient activity (MET ≥600). Sedentary time was divided into low, medium, and high levels based on tertiles. For missing variables in this study, we used different imputation methods for categorical and continuous variables with Python 3.10, and the code for imputation has been uploaded to GitHub. Detailed explanations of all variables can be found on the official NHANES website (https://www.cdc.gov/nchs/nhanes).

### 2.4 Statistical Analysis

On the basis of the multi‐stage probability sampling method in NHANES, all analyses were conducted with sample weighting, clustering, and stratification following the NHANES analytic guidelines (https://wwwn.cdc.gov/nchs/nhanes/analyticguidelines.aspx). The association between BRI and sleep patterns was evaluated using logistic regression by calculating the prevalence odds ratio (POR) and their 95% confidence intervals (CI). We constructed three multivariate regression models: a crude model (no adjustments), a minimally adjusted model (adjusted for age, race, and ethnicity), and a fully adjusted model (adjusted for all covariates). Restricted cubic splines (RCSs) were used to visualize the nonlinear relationship between BRI and sleep patterns. Furthermore, subgroup analyses of covariates of interest were conducted to assess the stability of the findings. Finally, ROC analysis was used to compare the performance of BRI and BMI in diagnosing unhealthy sleep patterns. All statistical analyses were performed using R 4.4.0 software and EmpowerStats, with the statistical significance level set at *p* < 0.05.

## 3 Results

### 3.1 Baseline Characteristics

A total of 23,236 participants were included in this study. Results showed that participants with unhealthy sleep patterns were more likely to be female, non‐Hispanic white, highly educated, married or cohabitating, with a PIR of 1.3–3.5, longer sedentary time, and without a history of CVD, hypertension, or diabetes, non‐drinkers but smokers (*p* < 0.05). Detailed information is presented in Table 1.

**TABLE 1:** Weighted demographic characteristics of all participants.

		Sleep pattern		
	Total	Healthy	Unhealthy	*p* value
Gender				<0.001
Male	10,374 (48.85%)	4966 (50.33%)	5408 (47.56%)	
Female	10,862 (51.15%)	4900 (49.67%)	5962 (52.44%)	
Age				0.777
<60	14,433 (67.96%)	6715 (68.06%)	7718 (67.88%)	
≥60	6803 (32.04%)	3151 (31.94%)	3652 (32.12%)	
Race				<0.001
Mexican American	3182 (14.98%)	1733 (17.57%)	1449 (12.74%)	
Other Hispanic	2167 (10.20%)	1036 (10.50%)	1131 (9.95%)	
Non‐Hispanic White	9289 (43.74%)	4322 (43.81%)	4967 (43.69%)	
Non‐Hispanic Black	4460 (21.00%)	1685 (17.08%)	2775 (24.41%)	
Other race	2138 (10.07%)	1090 (11.05%)	1048 (9.22%)	
Education				<0.001
Less than high school	5488 (25.84%)	2615 (26.51%)	2873 (25.27%)	
High school	4839 (22.79%)	2100 (21.29%)	2739 (24.09%)	
College or higher	10,909 (51.37%)	5151 (52.21%)	5758 (50.64%)	
Marital status				<0.001
Married/Living with a partner	12,601 (59.34%)	6146 (62.29%)	6455 (56.77%)	
Divorced/Widowed/Separated	4681 (22.04%)	1820 (18.45%)	2861 (25.16%)	
Never married	3954 (18.62%)	1900 (19.26%)	2054 (18.07%)	
Mean poverty income ratio				<0.001
<1.3	6468 (30.46%)	2813 (28.51%)	3655 (32.15%)	
1.3–3.5	8860 (41.72%)	4172 (42.29%)	4688 (41.23%)	
≥3.5	5908 (27.82%)	2881 (29.20%)	3027 (26.62%)	
CVD				<0.001
Yes	2135 (10.05%)	783 (7.94%)	1352 (11.89%)	
No	19,101 (89.95%)	9083 (92.06%)	10,018 (88.11%)	
Hypertension				<0.001
Yes	7481 (35.23%)	2918 (29.58%)	4563 (40.13%)	
No	13755 (64.77%)	6948 (70.42%)	6807 (59.87%)	
Diabetes				<0.001
Yes	2538 (11.95%)	955 (9.68%)	1583 (13.92%)	
No	18,698 (88.05%)	8911 (90.32%)	9787 (86.08%)	
metabolic equivalent (MET %)				0.250
<600	2981 (14.04%)	1414 (14.33%)	1567 (13.78%)	
≥600	18,255 (85.96%)	8452 (85.67%)	9803 (86.22%)	
Sedentary time				<0.001
Low	6161 (29.01%)	2993 (30.34%)	3168 (27.86%)	
Moderate	7239 (34.09%)	3315 (33.60%)	3924 (34.51%)	
High	7836 (36.90%)	3558 (36.06%)	4278 (37.63%)	
Alcohol consumption				0.069
Yes	15,871 (74.74%)	7316 (74.15%)	8555 (75.24%)	
No	5365 (25.26%)	2550 (25.85%)	2815 (24.76%)	
Smoking status				<0.001
Current	4525 (21.31%)	1719 (17.42%)	2806 (24.68%)	
Former	5034 (23.71%)	2265 (22.96%)	2769 (24.35%)	
Never	11,677 (54.99%)	5882 (59.62%)	5795 (50.97%)	

*Note*: Continuous variables are represented by mean ± SD, while categorical variables are denoted by (*n*, %).

Abbreviations: BRI, body roundness index; CVD, cardiovascular disease; PIR, mean poverty income ratio.

### 3.2 The Association Between BRI and Sleep Pattern

**FIGURE 2:**
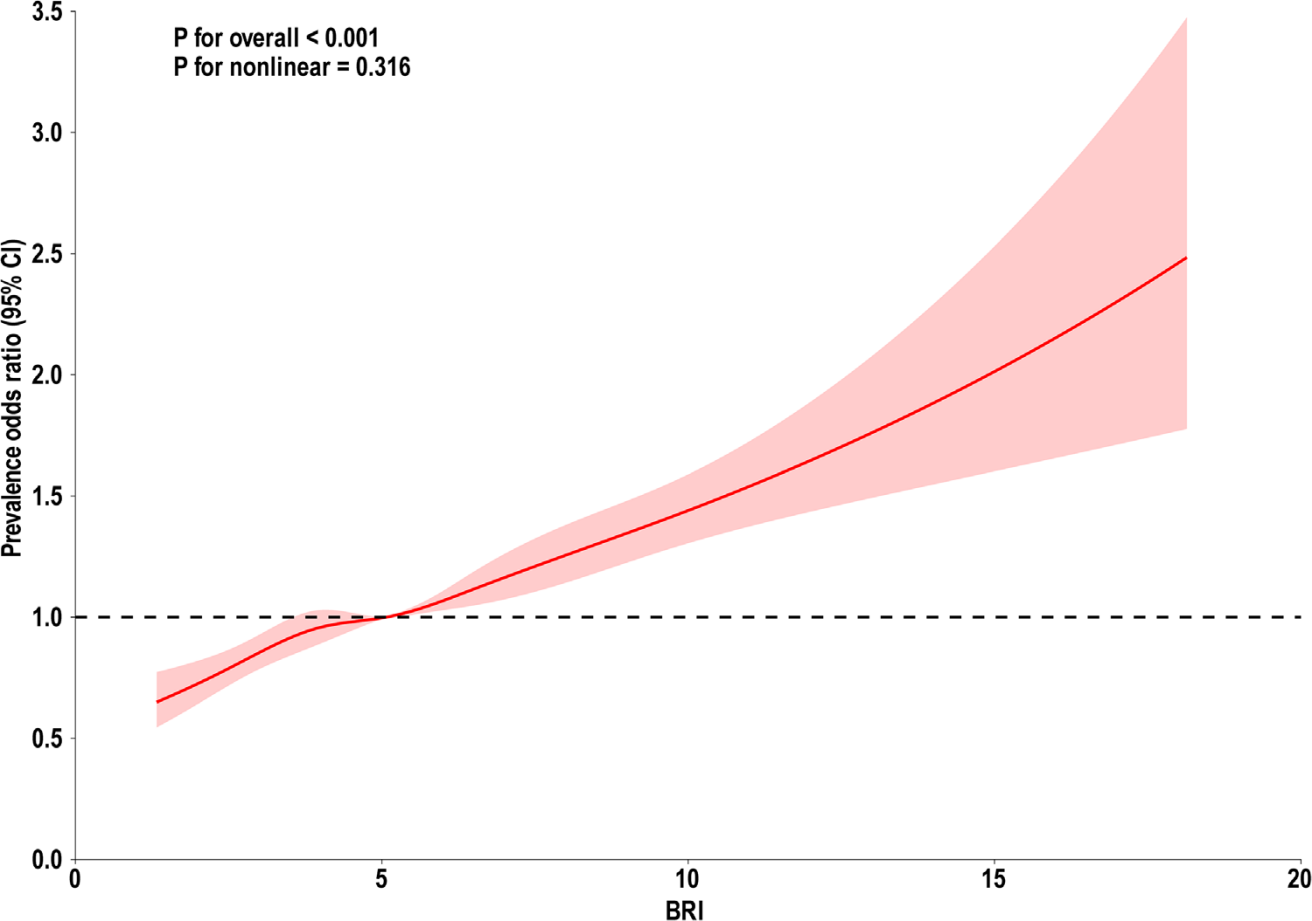
Associations between BRI levels and sleep patterns. CI, confidence interval.

Three models were constructed to examine the relationship between BRI and sleep patterns. This association was significant in the crude, minimally adjusted, and fully adjusted models. The crude model results showed that the POR (95% CI) was 1.11 (1.09–1.13), indicating that for each unit increase in BRI, the risk of unhealthy sleep patterns increased by 11%. In the minimally adjusted model, the POR (95% CI) was 1.12 (1.10–1.14), indicating a 12% increase in the risk of unhealthy sleep patterns for each unit increase in BRI. In the fully adjusted model, each unit increase in BRI was associated with a 9% increase in the risk of unhealthy sleep patterns (POR 1.09, 95% CI 1.07–1.10). In sensitivity analyses, when BRI was converted from a continuous variable to quartiles for trend testing, the results were consistent with the previous analyses. Using the first quartile (Q1) as the reference group, the results showed that the POR values for the second (Q2), third (Q3), and fourth quartiles (Q4) were higher than those for the Q1 group and were statistically significant across each model, as shown in Table 2. Furthermore, the RCS model indicated that the nonlinear relationship between BRI and unhealthy sleep patterns was not significant (Figure 2, overall *p* < 0.001, nonlinearity *p* > 0.05).

**TABLE 2:** Weighted multivariate logistic analysis: The association between body roundness index (BRI) levels and sleep pattern.

	Crude model		Minimally adjusted model		Fully adjusted model	
	POR (95% CI)	*p* value	POR (95% CI)	*p* value	POR (95% CI)	*p* value
BRI	1.11 (1.09, 1.13)	<0.0001	1.12 (1.10, 1.14)	<0.0001	1.09 (1.07, 1.10)	<0.0001
BRI quartile						
Q1	Ref.		Ref.		Ref.	
Q2	1.23 (1.11, 1.36)	0.0003	1.29 (1.16, 1.43)	<0.0001	1.23 (1.11, 1.37)	0.0003
Q3	1.36 (1.23, 1.51)	<0.0001	1.45 (1.31, 1.61)	<0.0001	1.34 (1.22, 1.48)	<0.0001
Q4	1.84 (1.66, 2.04)	<0.0001	1.93 (1.74, 2.16)	<0.0001	1.63 (1.46, 1.82)	<0.0001

*Note*: Crude model—no covariates are adjusted. Minimally adjusted mode—adjusted covariates include age, gender, and ethnicity. Fully adjusted model—adjusted covariates include age, gender, ethnicity, marital status, education levels, PIR, CVD, hypertension, diabetes, sedentary time, metabolic equivalent, smoking status, and alcohol consumption.

Abbreviations: CVD, cardiovascular disease; MET, metabolic equivalent; PIR, mean poverty income ratio.

### 3.3 Subgroup Analysis and Interaction Testing

**FIGURE 3:**
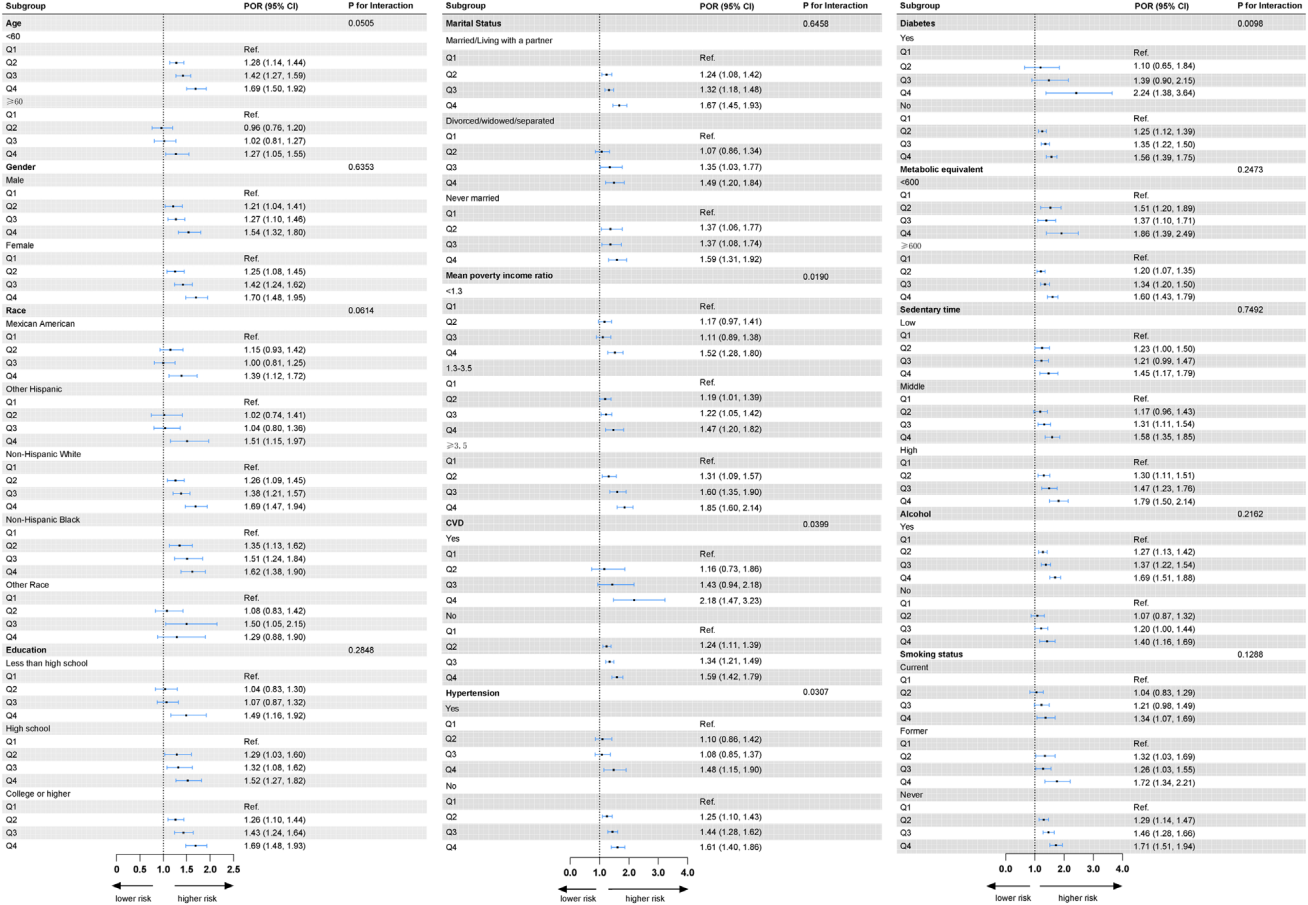
Subgroup analysis of associations between BRI levels and sleep patterns.

Figure 3 illustrates the results of the subgroup analyses, indicating that a higher BRI consistently maintained a positive association with unhealthy sleep patterns across most subgroups, further supporting the reliability of this study. Additionally, the subgroup analyses revealed that comorbidities such as CVD, hypertension, diabetes, and PIR may influence the relationship between BRI and sleep patterns.

In the PIR‐based subgroup analysis, the association between higher BRI and unhealthy sleep patterns varied across different income levels (P for interaction = 0.0190). In the group with PIR <1.3, BRI in Q4 was significantly associated with the risk of unhealthy sleep patterns, with a POR of 1.52 (95% CI: 1.28–1.80). For the group with PIR 1.3–3.5, the POR for Q4 was 1.47 (95% CI: 1.20–1.82), also showing a significant increase in risk. In the group with PIR ≥3.5, the POR for Q4 was the highest at 1.85 (95% CI: 1.60–2.14), indicating that the impact of BRI on unhealthy sleep patterns was most pronounced in the high‐income group.

Subgroup analysis based on CVD showed significant differences in the risk of unhealthy sleep patterns associated with higher BRI levels between individuals with and without CVD (P for interaction = 0.0399). Among patients with CVD, the POR for Q4 was 2.18 (95% CI: 1.47–3.23), suggesting a significantly increased risk of unhealthy sleep patterns in CVD patients with higher BRI. In contrast, among individuals without CVD, the POR for Q4 was 1.59 (95% CI: 1.42–1.79), indicating an increased risk, although to a lesser extent compared to those with CVD.

The subgroup analysis for hypertension showed significant differences in the effect of BRI levels on unhealthy sleep patterns between hypertensive and non‐hypertensive individuals (P for interaction = 0.0307). For patients with hypertension, the POR for Q4 was 1.48 (95% CI: 1.15–1.90), indicating a significant increase in risk. Among individuals without hypertension, the POR for Q4 was 1.61 (95% CI: 1.40–1.86), also showing a significant increase in the risk of unhealthy sleep patterns.

Subgroup analysis for diabetes indicated significant differences in the risk of unhealthy sleep patterns associated with BRI levels between diabetic and non‐diabetic individuals (P for interaction = 0.0098). Among diabetic patients, the POR for Q4 was 2.24 (95% CI: 1.38–3.64), suggesting that higher BRI levels significantly increased the risk of unhealthy sleep patterns. In non‐diabetic individuals, the POR for Q4 was 1.56 (95% CI: 1.39–1.75), indicating that the effect of BRI on unhealthy sleep patterns was less pronounced in the non‐diabetic population.

### 3.4 Comparison of BRI and BMI in Predicting

**FIGURE 4:**
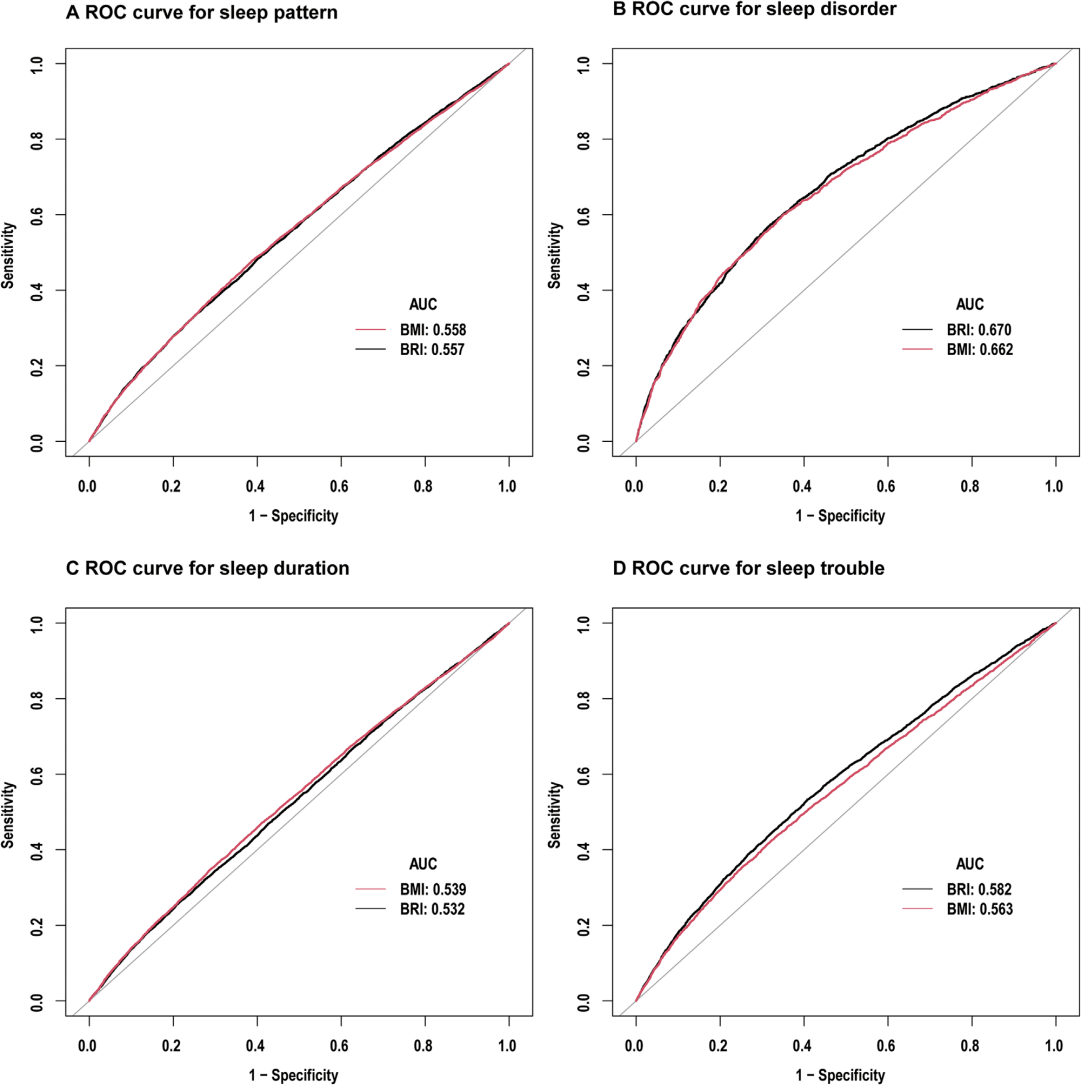
Comparison of BRI and BMI in predicting sleep pattern (A), sleep disorder (B), sleep duration (C), and sleep trouble (D). BMI, body mass index; BRI, body roundness index; ROC, receiver operating characteristic.

To compare the diagnostic effectiveness of BRI and BMI for unhealthy sleep patterns, we conducted diagnostic tests on sleep patterns and their components. For diagnosed sleep disorders, sleep trouble, and abnormal sleep duration, BRI generally outperformed BMI. However, the effectiveness of both BRI and BMI was comparable in diagnosing overall unhealthy sleep patterns, as detailed in Figure 4 and Table 3.

**TABLE 3:** Comparison of body roundness index (BRI) levels and body mass index (BMI) in predicting sleep pattern.

Comparison of ROC curves for BRI and BMI in predicting sleep pattern						
	AUC	Cut‐off	Specificity	Sensitivity	Accuracy	*p* for difference in auc
BRI	0.5573 (0.5496, 0.5650)	6.2814	0.7479	0.3345	0.5266	0.8674
BMI	0.5576 (0.5499, 0.5653)	28.8150	0.6119	0.4786	0.5406	

Comparison of ROC curves for BRI and BMI in predicting sleep disorder						
	AUC	Cut‐off	Specificity	Sensitivity	Accuracy	*p* for difference in auc
BRI	0.6695 (0.6561, 0.829)	5.9654	0.6794	0.5747	0.6705	0.0129
BMI	0.6625 (0.6488, 0.6761)	29.9450	0.6519	0.5976	0.6474	

Comparison of ROC curves for BRI and BMI in predicting sleep duration						
	AUC	Cut‐off	Specificity	Sensitivity	Accuracy	*p* for difference in auc
BRI	0.5317 (0.5238, 0.5396)	6.8089	0.7915	0.2554	0.5665	<0.0001
BMI	0.5391 (0.5312, 0.5470)	28.8250	0.5895	0.4709	0.5397	

Comparison of ROC curves for BRI and BMI in predicting sleep trouble						
	AUC	Cut‐off	Specificity	Sensitivity	Accuracy	*P* for difference in auc
BRI	0.5815 (0.5725, 0.5906)	5.3980	0.5924	0.5330	0.5778	<0.0001
BMI	0.5628 (0.5536, 0.5720)	30.3450	0.6784	0.4237	0.6158	

Abbreviation: ROC, receiver operating characteristic.

## 4 Discussion

In this study, we observed a positive correlation between BRI and the risk of unhealthy sleep patterns, and this association remained stable even after adjusting for covariates. Subsequent RCS analysis further highlighted the linear relationship between BRI and sleep patterns. Therefore, the findings suggest that BRI is a potential marker for assessing sleep patterns.

Many epidemiological studies have explored the association between sleep and obesity, but the conclusions remain divided. There are numerous indicators of obesity, with BMI being the most widely used in recent years to represent overall obesity (Wang et al. 2021). Several studies have shown that obesity is associated with abnormal sleep duration and poor sleep quality, which aligns with our findings (Wang et al. 2023). A longitudinal study from Australia indicated that both abnormal sleep duration and poor sleep quality are linked to an increased risk of obesity in adults (Keramat et al. 2023). In women, obesity is associated with self‐reported sleep duration and/or insomnia symptoms, but this correlation is not significant in men (Silva‐Costa et al. 2020). The discrepancies in study conclusions may be attributed to the diversity of study populations, differences in age groups, and the influence of other potential confounding factors. In our study, we found a positive correlation between BRI and the risk of unhealthy sleep patterns. As a measure of body fat distribution, BRI typically indicates higher visceral fat accumulation, which secretes various pro‐inflammatory factors, such as tumor necrosis factor‐α and interleukin‐6 (de Queiroz et al. 2024; Cossins et al. 2023). These pro‐inflammatory factors induce a chronic inflammatory state that can affect the central nervous system, particularly the parts that regulate the sleep–wake cycle, making individuals more prone to sleep disorders, difficulty falling asleep, or insufficient sleep duration (Irwin, Olmstead, and Carroll 2016). Besides, high BRI levels are also closely associated with a higher incidence of mental health issues. Studies have shown that psychological problems, such as depression and anxiety, are more common in individuals with high BRI, and these mental health issues can further disrupt sleep (Luo et al. 2024), leading to insomnia, frequent awakenings during the night, and other sleep disturbances (Zhang et al. 2024; Mason and Harvey 2014). Additionally, obesity, especially abdominal obesity, is a major factor leading to sleep breathing disorders, such as obstructive sleep apnea (OSA) (D'Angelo et al. 2023). As BRI increases, the risk of unhealthy sleep patterns also rises, potentially triggering a series of cascading health problems.

In this study, BRI demonstrated a stronger predictive ability than BMI in assessing certain aspects of sleep patterns. As an emerging indicator, BRI reflects body roundness related to height and waist circumference and provides a more accurate estimation of visceral fat levels compared to traditional anthropometric indices (Chang et al. 2015; Kiremitli et al. 2021). Studies have indicated that BRI can effectively predict arterial stiffness and metabolic syndrome (Zhang et al. 2017; Wu et al. 2021). Moreover, BRI shows a U‐shaped association with all‐cause mortality (Zhang et al. 2024). Thus, as an indicator that reflects body fat and visceral fat levels, BRI has the potential to become a new tool for health screening and disease risk prediction.

Our subgroup analysis indicates that comorbidities and PIR may influence the association between BRI and sleep patterns. CVD patients often experience chronic inflammation and metabolic abnormalities, which can amplify the negative impact of BRI on sleep (Battineni et al. 2021; Min et al. 2024). For example, obesity and poor body fat distribution can exacerbate inflammatory responses, further affecting sleep quality (Gaines et al. 2016). Additionally, individuals with these conditions may have poorer overall health, making a higher BRI significantly increase their physical burden, leading to more severe sleep issues (Bishop et al. 2023). Although an association between BRI and unhealthy sleep patterns was also observed in the non‐CVD population, this association was relatively weaker, suggesting that in healthier individuals, BRI still affects sleep but to a lesser extent compared to those with CVD. For patients with hypertension, higher BRI levels significantly increase the risk of unhealthy sleep patterns. High BRI may affect blood pressure regulation mechanisms, leading to greater blood pressure fluctuations, which are often associated with decreased sleep quality (Luque‐Ramírez et al. 2007; Javaheri et al. 2008). Hypertensive individuals may have an overactive sympathetic nervous system, closely bound to obesity or poor fat distribution, further impacting sleep quality (Esler, Osborn, and Schlaich 2024; Hillebrand et al. 2014). In individuals without hypertension, BRI is slightly associated with the risk of unhealthy sleep patterns. This could be due to other pathways through which BRI affects sleep, such as sleep apnea caused by obesity (Allam et al. 2007). Diabetic patients often face issues like insulin resistance and glucose metabolism abnormalities, which higher BRI levels can exacerbate, making them more prone to sleep trouble (Tenda et al. 2024). These individuals are more likely to experience sleep disorders such as sleep apnea, which are closely related to body fat distribution and obesity (Tenda et al. 2024). Among non‐diabetic individuals, BRI is still associated with unhealthy sleep patterns, though the association is milder. This may be because non‐diabetic individuals have better metabolic conditions, making BRI's impact on sleep less pronounced. Higher BRI implies greater abdominal fat accumulation, associated with an increased risk of hypertension, atherosclerosis, myocardial infarction, and other CVDs (Casanueva et al. 2010). Long‐term unhealthy sleep patterns, such as OSA and insomnia, are closely related to CVDs. In turn, unhealthy sleep patterns can further exacerbate these disease risks, creating a vicious cycle (Jike et al. 2018). In patients with CVD, hypertension, or diabetes, careful management of BRI levels may be necessary to reduce the risk of unhealthy sleep patterns. For low‐ and middle‐income individuals, factors like poor quality of life, unhealthy diets, and worse living conditions significantly impact their sleep, potentially overshadowing the influence of BRI (Rodríguez‐Modroño and López‐Igual 2021; Wakata, Nishioka, and Takaki 2022; Deng et al. 2024). High‐income (PIR ≥3.5) groups may face greater life pressures, such as long working hours and sedentary lifestyles (Shuval, Li, and Gabriel 2017). These individuals often have better access to healthcare resources and greater health awareness; thus, a high BRI may serve as a serious warning signal, strongly affecting their sleep (Yang et al. 2010).

This study has several limitations. First, due to the cross‐sectional design, it is impossible to establish a causal relationship between BRI and sleep patterns. Future longitudinal studies are needed to enhance the reliability of the findings. Second, the data on sleep patterns were self‐reported. Research has shown that there are differences between self‐reported sleep difficulties and perceived sleep duration and objectively measured sleep duration obtained through actigraphy (Ding et al. 2022). Moreover, recall bias may lead to information inaccuracies in reported sleep duration (Zhang et al. 2019). Although self‐reported methods offer representative insights into sleep patterns in large‐scale population studies, they may not always align with objective measurements. Despite scientifically validated sleep assessment tools can provide accurate data on sleep structure, self‐reported questionnaires are commonly used to evaluate sleep health due to their cost‐effectiveness and ease of application on a large scale (Bothelius et al. 2015; Jung et al. 2017). Lastly, other potential confounding factors might not have been fully considered in this study. Despite these limitations, this remains a population‐based study aimed at exploring the relationship between BRI and unhealthy sleep patterns among US adults (Tamhane et al. 2016).

In conclusion, our study demonstrates a significant positive correlation between BRI and unhealthy sleep patterns. This finding is crucial for understanding the relationship between obesity and sleep health, suggesting that BRI may serve as a potential indicator for assessing sleep health.

## Author Contributions

**Pingchuan Liu**: conceptualization, methodology, validation, writing–original draft, writing–review and editing. **Yuding Luo**: conceptualization, methodology, writing–original draft, writing–review and editing. **Xing He**: methodology, software, validation. **Jiali Zhang**: supervision. **Fanzhou Ren**: formal analysis, supervision. **Bingyang Zhang**: investigation. **Bo Zheng**: validation. **Jian Wang**: funding acquisition.

## Ethics Statement

The data for this study was obtained from the NHANES public database, and participants in the survey had already received and signed an informed consent form. Therefore, this study is exempt from the need for consent to participate.

## Consent

The authors have nothing to report.

## Conflicts of Interest

The authors declare no conflicts of interest.

### Peer Review

The peer review history for this article is available at https://publons.com/publon/10.1002/brb3.70224

## Data Availability

The data for this study were obtained from the public database NHANES. More comprehensive raw data can be accessed and downloaded from the official website of the NHANES database (https://www.cdc.gov/nchs/nhanes/index.htm).
